# DO INTRA-PROXY GAPS EMERGE WHEN FAMILY CAREGIVERS ASSESS QOL FROM DIFFERENT PERSPECTIVES?

**DOI:** 10.1093/geroni/igz038.3547

**Published:** 2019-11-08

**Authors:** Patricia Egan

**Affiliations:** University of Wisconsin-Madison, Madison, Wisconsin, United States

## Abstract

Dementia family caregivers are routinely enlisted as proxy assessors of care recipients’ quality of life (QOL). This study explored whether prompts to change perspective during QOL assessment could elicit an intra-proxy gap. The intra-proxy gap was hypothesized to be any difference between those assessments made from the caregiver’s own perspective and those made from the adopted perspective of the care recipient, as the care giver imagined it to be (Pickard and Knight, 2005). Thirty-six dementia family caregivers were recruited from senior service agencies. Subjects completed the Quality of Life-Alzheimer Disease (QOL-AD), Caregiver Version under two conditions: First, from an unprompted perspective and second, from the adopted perspective of the care recipient, as the family caregiver imagined it to be. T-testing indicated intra-proxy gaps emerged for eleven of the QOL-AD’s thirteen domains. For these domains, QOL scores were higher when assessed from the care recipient’s perspective, as the family caregiver imagined it to be. The sample was then repeatedly bisected using caregivers’ personal, relational, and health factors. T-testing indicated that family caregivers’ personal factors were associated with intra-proxy gaps across more QOL-AD domains than their relational or health factors were. Three personal factors, being of older age, having more empathetic concern, and having more empathetic distress, were associated with intra-proxy gaps more frequently than other personal factors were. Findings suggest that clinicians should be alert for perspective employed by proxy assessors and for the possibility of intra-proxy gaps. Recognition of these gaps could help improve interpretation of QOL scores.

